# Impact of Contrast-Enhanced Mammography on Personalized Surgical Decision-Making in Ductal Carcinoma In Situ: A Multicentre Pilot Observational Cohort Study

**DOI:** 10.3390/jpm16070383

**Published:** 2026-07-17

**Authors:** Petra Valković Zujić, Nina Bartolović, Manuela Avirović, Emina Babarović, Lucija Požgaj, Maja Prutki, Emina Grgurević Dujmić, Ana Car Peterko

**Affiliations:** 1 Department of Diagnostic and Interventional Radiology, Clinical Hospital Centre Rijeka, Kresimirova 42, 51000 Rijeka, Croatia; 2 Department of Radiology, Faculty of Medicine, University of Rijeka, Brace Branchetta 20, 51000 Rijeka, Croatia; 3 Department of Pathology, Faculty of Medicine, University of Rijeka, Brace Branchetta 20, 51000 Rijeka, Croatia; 4 Department of Pathology, Clinical Hospital Centre Rijeka, Kresimirova 42, 51000 Rijeka, Croatia; 5 Department of Diagnostic and Interventional Radiology, University Hospital Centre Zagreb, Kišpatićeva 12, 10000 Zagreb, Croatia; 6 School of Medicine, University of Zagreb, Šalata 3, 10000 Zagreb, Croatia; 7 Community Health Centre Primorsko-Goranska County, Kresimirova 52A, 51000 Rijeka, Croatia; 8 Department of General Surgery and Surgical Oncology, Clinical Hospital Centre Rijeka, Kresimirova 42, 51000 Rijeka, Croatia

**Keywords:** ductal in situ carcinoma, calcifications, breast surgery, screening, contrast enhanced mammography

## Abstract

**Background:** Accurate delineation of ductal carcinoma in situ (DCIS) remains a key challenge in surgical planning. Although contrast-enhanced mammography (CEM) improves lesion detection, its clinical role in informing individualized surgical strategies remains unclear. This study evaluated the impact of CEM on preoperative assessment and surgical planning, with particular emphasis on whether its effect varies across patient subgroups. **Methods:** This multicentre pilot observational cohort study included 102 patients: 51 prospective patients undergoing preoperative CEM and mammography (MMG) and 51 retrospective controls assessed with MMG alone. Imaging-derived lesion size and planned resection volume were compared with pathological size using correlation analysis, intraclass correlation coefficient (ICC), and Bland–Altman methods. Surgical outcomes were assessed, and subgroup analyses explored differences according to CEM enhancement status. **Results:** CEM showed improved correlation with pathological DCIS size compared with MMG (ρ = 0.54 vs. 0.37), with the strongest agreement in CEM-positive lesions (ρ = 0.67; ICC 0.745). However, its clinical impact was not uniform. At the population level, CEM did not significantly change planned resection volume or surgical thresholds. In contrast, in CEM-positive patients, CEM was associated with larger planned resections, proportional to pathological tumor burden. Reoperation rates were lower in the CEM cohort (5.9% vs. 17.6%), without statistical significance, and margin status was comparable. **Conclusions:** The impact of CEM on surgical planning in DCIS is heterogeneous and largely confined to patients with enhancing lesions. These findings suggest that the value of CEM may lie in its selective use, where it can refine assessment of disease extent in specific subgroups rather than in routine application. Further studies incorporating predictive approaches are needed to support risk-adapted imaging strategies.

## 1. Introduction

Ductal carcinoma in situ (DCIS) is the earliest form of malignant lesion in the breast, which in most cases is diagnosed by mammography screening, usually in the form of asymptomatic calcifications. The question of whether DCIS is a true malignancy of the breast, which pathological criteria are used to diagnose and classify DCIS, and the questions of the nature of the disease and its overtreatment are controversial.

Surgery is still the primary treatment of DCIS, and the status of the surgical margins is of paramount importance, because residual microscopic disease at the margin is the principal risk factor for ipsilateral breast tumor recurrence for both DCIS and invasive cancer, and because surgical excision may also reveal an upgrade to invasive carcinoma [1,2].

These controversies influence the rationale for preoperative imaging, as accurate assessment of disease extent is critical for surgical planning and minimizing overtreatment [1,3]. Breast-conserving surgery (BCS) is preferred for most patients with localized DCIS, while mastectomy is reserved for extensive, multicentric, or anatomically challenging lesions, or when negative margins cannot be achieved [1].

Compared to invasive ductal carcinoma, the re-excision rate in DCIS is relatively high (30–40%) [4,5]. A high re-excision rate can be attributed to the fact that DCIS is often non-palpable and radiologically ill-defined, with microscopic spread beyond what is visible on imaging, making complete excision challenging even when aiming for the recommended ≥2 mm margin. Segmental distribution, larger or more extensive lesions, and discrepancies between radiological and pathological size estimates further increase the likelihood of close or involved margins, as do patient- and tumor-related factors such as younger age, high nuclear grade, premenopausal status, and hormone receptor negativity [6,7].

The latest evidence-based strategies to reduce re-excision rates in patients with DCIS include advances in imaging and evolving surgical guidelines. Due to high upgrade rates to invasive carcinoma, florid and pleomorphic lobular carcinoma in situ (LCIS) are also considered clinically significant in situ lesions and should be treated similarly to DCIS [8].

Because the diagnosis of DCIS is closely associated with mammographic detection of pathologic calcifications, it was assumed that magnetic resonance imaging (MRI) of the breast would provide little or no value for their detection and visualization [3,9]. However, a study conducted by Kuhl et al. showed that breast MRI has a significantly higher sensitivity than mammography in detecting DCIS [10].

In recent years, mammography with an iodine contrast agent, known as contrast-enhanced mammography (CEM), has been introduced, which, like MRI, is based on the evaluation of tumor angiogenesis [11]. It is important to emphasize that the sensitivity of CEM in detecting malignant lesions corresponds to the sensitivity of MRI [12,13,14]. Compared to MRI, CEM has numerous advantages, including lower costs, shorter image acquisition times, shorter time required to analyse the mammogram and read the findings, better patient acceptance and tolerance, and, importantly, wider availability. It is also a preferred imaging modality for patients with claustrophobia or specific contraindications to MRI [12,15,16]. Furthermore, a study by Hobbs shows that CEM is better tolerated by patients than MRI [17]. In addition to the contrast enhancement of breast lesions, CEM can also visualize pathological calcifications that are not visible on MRI, as well as overcome some of the limitations of conventional mammography, particularly in women with dense breast tissue, where lesion conspicuity may be reduced.

However, CEM also has several important limitations. Because DCIS generally exhibits lower levels of angiogenesis than invasive breast cancer, some lesions may demonstrate little or no enhancement, potentially limiting lesion conspicuity on recombined images. In addition, CEM requires intravenous administration of an iodinated contrast agent, which carries a small risk of allergic reactions and exposes patients to a slightly higher radiation dose than conventional mammography. Other disadvantages include the lack of three-dimensional visualization, the need for breast compression, and limited assessment of the axilla. Furthermore, when a lesion is visible only on CEM and cannot be identified on ultrasound or conventional mammography, tissue sampling may require CEM-guided biopsy, a technique that depends on specialized equipment and expertise that may not be universally available [12,14,15].

Recent studies suggest that CEM may improve the estimation of disease extent and influence surgical planning; however, its impact on clinically relevant outcomes such as margin status and reoperation rates remains inconsistent [18,19]. Importantly, current imaging strategies are primarily focused on lesion detection and size estimation, with limited integration into individualized clinical decision-making.

This gap highlights a critical unmet need in DCIS management: the translation of imaging findings into actionable, patient-specific surgical strategies that go beyond lesion detection alone.

In this context, there is increasing interest in imaging approaches that support personalized management of DCIS by identifying patient subgroups in whom more or less extensive surgery may be appropriate. Rather than applying imaging uniformly, such strategies aim to tailor surgical planning based on lesion-specific characteristics, thereby balancing the risks of overtreatment and undertreatment.

Accordingly, the clinical value of advanced imaging modalities such as CEM may lie not only in improved diagnostic accuracy but also in their ability to stratify patients and guide individualized treatment pathways.

Therefore, the primary research question of this study was whether preoperative CEM improves the estimation of DCIS extent and influences surgical planning compared with standard mammography. We hypothesized that CEM would demonstrate stronger agreement with pathological DCIS size than mammography alone and would provide additional information relevant to surgical decision-making. We further hypothesized that the clinical impact of CEM would not be uniform across all patients but would be most pronounced in patients with enhancing lesions, supporting a more individualized and risk-adapted approach to surgical planning.

## 2. Materials and Methods

### 2.1. Study Population

The study was conducted in accordance with the Declaration of Helsinki and approved by the Ethical Committee of Community Health Centre Primorsko-Goranska County, Centre for Prevention and Diagnosis of Chronic Diseases, Rijeka, Croatia (20 December 2024; approval number 01-86/5-2-24). The Community Health Centre Primorsko-Goranska County, Centre for Prevention and Diagnosis of Chronic Diseases, served as the coordinating institution for the study, while surgical treatment and pathological assessment were performed at collaborating tertiary care hospitals as part of routine clinical care. Following Institutional Review Board approval, we conducted a multicentre observational pilot cohort study consisting of a prospective CEM cohort and a retrospective control cohort. The study comprised a prospective cohort enrolled between 2024 and 2025 and a retrospective control cohort identified from patients treated between 2019 and 2024. The prospective CEM cohort included 51 consecutive patients with newly diagnosed DCIS, pleomorphic lobular carcinoma in situ (pLCIS), or DCIS with microinvasion. These patients provided written informed consent to participate in the study and to undergo CEM in addition to standard preoperative mammography. They subsequently underwent surgery at Clinical Hospital Centre Rijeka (CHC) and University Hospital Centre (UHC) Zagreb between 2024 and 2025. The retrospective control cohort consisted of patients diagnosed with DCIS who underwent surgery at CHC Rijeka between 2019 and 2024. Their clinical data were obtained from the prospectively maintained clinical register for breast diseases at CHC Rijeka and the Integrated Hospital Informatics System (IBIS). The Institutional Review Board waived the requirement for patient consent for this cohort due to the retrospective nature of the data collection.

#### 2.1.1. Inclusion Criteria

Study inclusion required a pathohistologically confirmed diagnosis of either pure ductal carcinoma in situ (DCIS) or DCIS with microinvasion. Additionally, two cases of pleomorphic lobular carcinoma in situ (pLCIS) without an invasive component were included (these entities were included due to their clinical similarity to DCIS in the preoperative setting and present comparable challenges in imaging-based assessment of disease extent). These diagnoses were established preoperatively via vacuum-assisted breast biopsy (VABB) or ultrasound-guided core needle biopsy (CNB). All cases were reviewed by the multidisciplinary breast teams at the Clinical Hospital Center (CHC) Rijeka or the University Hospital Centre (UHC) Zagreb, followed by surgical intervention at one of these two institutions.

#### 2.1.2. Exclusion Criteria

Patients were excluded from the study if they met any of the following criteria: clinical contraindications for CEM (including documented renal insufficiency, iodine allergy, hyperthyroidism, pregnancy, or breastfeeding); a history of prior breast interventions or imaging (specifically preoperative breast MRI, ipsilateral DCIS recurrence, or previous surgery for invasive carcinoma in the same breast or the presence of invasive carcinoma in the same breast), or the presence of invasive carcinoma (except for foci of microinvasion (<1 mm) in the preoperative or final pathohistological report). Additionally, patients were excluded if CEM examinations were technically inadequate for diagnostic interpretation due to positioning errors, contrast medium extravasation, or subtraction artifacts.

**Figure 1:**
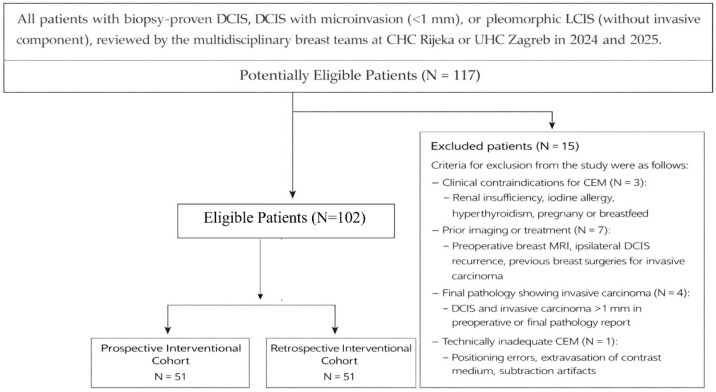
Standards of Reporting of Diagnostic Accuracy (STARD 2015) flowchart of patients included in the study.

The flow chart of patients included in the study is based on the Standards for Reporting of Diagnostic Accuracy (STARD 2015) guidelines (Figure 1) [20].

#### 2.1.3. Clinicopathological Data

Clinicopathological data, including age at diagnosis, tumor laterality, mode of detection, mammographic BI-RADS classification, mammographic DCIS size, clinical examination findings, hormone receptor status (estrogen receptor [ER] and progesterone receptor [PR]), Ki-67 proliferative index, final pathological diagnosis including type of in situ disease, nuclear (histological) grade and pathological DCIS size, time interval from initial MMG to surgery, surgical specimen size, surgical procedure, reoperation status, and surgical margin status, were obtained from institutional patient’s medical records. Due to institutional differences in pathological reporting, several variables contained missing data. Ki-67 proliferative index was not routinely assessed for DCIS patients at the University Hospital Centre Zagreb, resulting in missing Ki-67 data for 15 patients. Pathological DCIS size was unavailable for two patients (one from each cohort). Nuclear grade was missing in three patients from the control cohort. Surgical margin status was missing in three patients (one in the control cohort and two in the prospective cohort).

Surgical margin status was classified according to the distance between DCIS and the inked surgical margin. A positive surgical margin was defined as the presence of DCIS on the inked margin. Positive margins were further classified as focal, defined as a single microscopic focus of DCIS touching the inked margin with a length ≤ 2 mm, or wide, defined as either multiple foci of DCIS touching the inked margin or a single focus with a length > 2 mm. A negative free margin was defined as DCIS located ≥2 mm from the inked margin, while a close margin was defined as DCIS located <2 mm from the margin but not on ink.

Reoperation was considered in cases with positive surgical margins. However, surgical management strategies evolved during the study period. In the historical control cohort (2019–2024), reoperation was generally considered for patients with close margins as well as focal or wide positive margins. In contrast, in the prospective cohort (2024–2025), reoperation was primarily recommended for patients with wide positive margins, while patients with close or focal margins were considered for reoperation selectively, particularly in younger patients (<45 years), those with high nuclear grade (NG3), or hormone receptor–negative tumor biology.

All clinicopathological characteristics of both cohorts and their comparison are summarized in Table 1.

### 2.2. Data Analysis and Image Interpretation

Demographic data and morphological characteristics of the CEM findings were recorded, including the presence or absence of lesions, lesion size (measured in millimetres), and assigned BI-RADS category. Two radiologists specializing in breast imaging, with 22 and 8 years of experience in mammography interpretation, respectively, evaluated the morphological features of both standard digital mammography (MMG) and CEM through a consensus reading. The extent of disease was assessed in accordance with the BI-RADS lexicon for contrast-enhanced mammography [21]. To standardize analysis, only one lesion per breast was recorded; in cases of multifocal or multicentric findings, the maximum overall diameter of the suspicious area was utilized for measurements.

**Figure 2:**
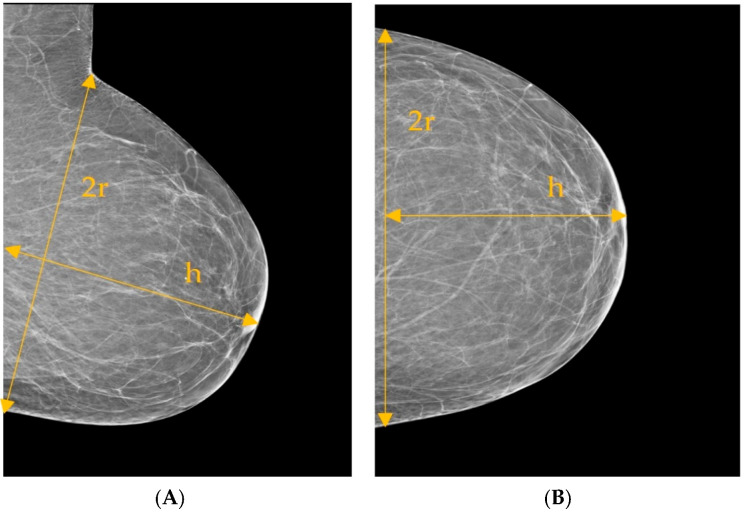
Mediolateral oblique (**A**) and craniocaudal (**B**) mammographic projections of the left breast, illustrating the standardized breast measurements utilized in this study.

Breast dimensions were measured in centimetres using standard mediolateral oblique (MLO) and craniocaudal (CC) mammographic projections (Figure 2). On the MLO projection, the longitudinal breast dimension (2r) was defined as the distance from the inframammary fold to the superior border of the pectoralis major muscle. The anteroposterior diameter (h) was defined as the distance from the nipple to the pectoralis major muscle on the MLO projection and from the nipple to the pectoralis muscle or posterior image margin on the CC projection. The breast diameter (2r) on the MLO view was measured from the inframammary fold to the superior aspect of the breast. On the CC view, the 2r measurement represents the transverse distance from the medial to the lateral breast margins in the posterior region of the projection.

CEM positivity was defined as the visualization of pathologically significant enhancement. Rigorous interpretation criteria were applied to differentiate suspicious findings from background parenchymal enhancement (BPE), ensuring that only areas of focal or non-mass enhancement distinct from normal physiological uptake were categorized as pathologic.

### 2.3. CEM Protocol

Before undergoing CEM, each patient completed and signed the Patient Referral Questionnaire for examinations involving an iodine contrast agent, as well as the Consent for the examination and participation in the study. After verifying medical histories and confirming adequate renal function, an intravenous access via the antecubital vein was placed. Contrast administration was performed using an automated injector at a rate of 2–3 mL/s, delivering a weight-based dose of 1.5 mL/kg (300–370 mgI/mL). This was immediately followed by a 20 mL saline flush at an identical flow rate to optimize contrast distribution and subsequent image quality. Patient safety was prioritized through continuous monitoring for adverse reactions, with venous access maintained for the duration of the study.

Imaging commenced two minutes post-injection with the patient in the standard mammographic position. The protocol required bilateral low- and high-energy acquisitions in both craniocaudal (CC) and mediolateral oblique (MLO) projections. To capture peak enhancement and mitigate the risk of false negatives due to early contrast washout, imaging always originated with the breast containing the suspicious finding.

### 2.4. Imaging Acquisition

All the images in Clinical Hospital Centre were taken on the Selenia Dimensions Mammography System, Marlborough, MA, USA, and contrast-enhanced mammography on Siemens Mammomat Revelation, Siemens Healthineers, Erlangen, Germany.

All the images in University Hospital Centre Zagreb were taken on Selenia Senographe Pristina GE Healthcare, Chicago, IL, USA, Selenia 9000 Hologic, Marlborough, MA, USA, and Planmed clarity 3D, Planmed, Roselle, IL, USA.

Low-energy acquisitions utilized a voltage range of 26–33 kVp, consistent with standard digital mammography, employing either rhodium or tungsten filtration. In contrast, high-energy images were acquired at a higher setting of 49 kVp using a combination of titanium and tungsten filters. By subtracting the background glandular tissue, the system produced recombined images that, along with the low-energy views, were then transferred to the picture archiving and communication system (PACS) [17].

### 2.5. Surgical Planning and Complexity

Percentage of breast resection volume based on MMG findings and CEM findings, calculated using formula 1 for each patient in the intervention cohort [22]:

(4 × (lesion radius + 1 cm)^3^) ÷ (breast radius^2^ × breast projection) (1)

All sizes are determined from mammography findings according to the scheme shown earlier in Figure 2.

The extent of resection relative to the total breast volume served as a primary determinant for surgical complexity. A resection volume exceeding 15% was considered an indication for advanced oncoplastic techniques (second and third degree) or total mastectomy with immediate reconstruction and sentinel lymph node biopsy (SLNB). To evaluate healthcare efficiency and surgical wait times, the interval from the initial clinical or radiological consultation at Clinical Hospital Center (CHC) Rijeka to the definitive surgical procedure was calculated and compared for patients in both study groups.

The authors claim that generative artificial intelligence (GenAI) has not been used in this paper to generate text, data, or graphics, or to assist in study design, data collection, analysis, or interpretation.

The study was reported on the ClinicalTrials.gov platform under the number NCT06217458.

### 2.6. Statistical Analysis

Continuous variables are presented as medians with ranges or interquartile ranges (IQR), and categorical variables as counts and percentages. Given the small sample size and significant deviations from normality (D’Agostino–Pearson test, *p* < 0.05) of multiple continuous variables, non-parametric statistical methods were considered more appropriate and robust than parametric alternatives.

Comparisons between independent groups were conducted using the Mann–Whitney U test for continuous variables and the χ^2^ test or Fisher’s exact test for categorical variables, as appropriate. Trends across ordered categories were assessed using the χ^2^ test for trend.

To compare DCIS imaging-derived measurements and pathological DCIS size (reference standard), several complementary methods were used: Spearman’s rank correlation coefficient (ρ), intraclass correlation coefficient (ICC), absolute error comparison using the paired Wilcoxon signed-rank test, and the non-parametric Bland–Altman method. In Bland–Altman analysis, bias was reported as the median difference between imaging-derived and pathological DCIS size, and limits of agreement (LoA) were defined by the 2.5th and 97.5th percentiles of the differences.

For comparisons of DCIS size estimates obtained by mammography, contrast-enhanced mammography (CEM), and pathology within the same patients, the Friedman test for related samples was used. When the Friedman test indicated a statistically significant overall difference, post hoc pairwise comparisons were performed using the Conover test.

Linear regression analysis was performed to assess the relationship between planned resection volume and pathological DCIS size. Results were reported using the correlation coefficient (r), coefficient of determination (R^2^), and corresponding *p* values.

Paired dichotomous outcomes, including classification of surgical complexity (≤15% vs. >15% resection volume) based on mammography and contrast-enhanced mammography within the same patients, were compared using McNemar’s test. For analyses involving the prospective CEM cohort, results were evaluated in the full cohort and in the subgroup of CEM-positive patients. Additional analyses were conducted to evaluate the consistency of imaging–pathology correlations across subgroups defined by contrast enhancement, reflecting a personalized approach to imaging-based surgical decision-making.

Because this was a non-randomized observational study with a retrospective control cohort, additional analyses were performed to evaluate the potential influence of selection bias, confounding, and temporal bias.

No formal a priori sample size calculation was performed because this study was designed as a pilot feasibility study. A post hoc sample size estimation based on the observed reoperation rates was performed using Fisher’s exact test in MedCalc^®^ (version 23.3.4).

Multivariable logistic regression was used to assess the association between study group and reoperation, adjusting for age, nuclear grade, and treatment center.

To further address potential confounding, a propensity score representing the probability of assignment to the prospective cohort was estimated using logistic regression, including age, nuclear grade, and treatment center and was included as a covariate in the outcome model.

A sensitivity analysis was performed by restricting the control group to recent patients only, defined as those enrolled between 2022 and 2023, to minimize potential temporal bias.

Due to the limited number of outcome events (N = 12), multivariable models were at risk of overfitting. Therefore, a propensity score approach was additionally used to reduce model dimensionality.

A two-sided *p* value < 0.05 was considered statistically significant. All analyses were performed using MedCalc for Windows, version 23.3.4 (MedCalc Statistical Software bvba, Ostend, Belgium).

## 3. Results

### 3.1. Control Cohort of Patients (MMG vs. Pathology)

In the control cohort (N = 50), the median DCIS size measured by MMG was 11 mm (range 2–50 mm), compared with 14 mm (range 1–70 mm) on pathological examination. The median paired difference was 3 mm (Hodges–Lehmann estimate) with a trend toward larger sizes on pathology: there were more positive than negative differences (27 vs. 20), but this difference was not statistically significant (Wilcoxon signed-rank test, *p* = 0.15). All other conducted analyses of agreement between MMG and pathology in the control cohort of patients are shown in Table 2.

### 3.2. Prospective CEM Cohort: MMG, CEM, and Pathology

In the overall prospective cohort (N = 50), including both CEM-positive and CEM-negative patients, the median DCIS size was 16 mm (IQR 11–30) on mammography, 4.5 mm (IQR 0–25) on CEM, and 15 mm (IQR 7–25) on pathology. A Friedman test demonstrated a statistically significant difference among the three measurement methods (*p* = 0.038). Post hoc pairwise comparisons showed a significant difference between mammography and CEM measurements, whereas neither modality differed significantly from pathological size.

When the analysis was restricted to CEM-positive cases (N = 25), the median DCIS size was 22 mm (IQR 13.3–41) on mammography, 25 mm (IQR 17.3–65.3) on CEM, and 25 mm (IQR 10–46.3) on pathology. The Friedman test again demonstrated a statistically significant difference among the three measurement methods (*p* = 0.0047). Post hoc comparisons indicated that CEM measurements differed significantly from both mammography and pathology, while mammography did not differ significantly from pathology.

To further evaluate the relationship and agreement between imaging-derived and pathological DCIS size, Spearman correlation, ICC, and Bland–Altman analysis were performed. The results for the entire prospective cohort and only the CEM-positive subgroup are detailed in Table 2.

### 3.3. Method Comparison and Agreement with Pathological DCIS Size

The relationship and agreement between imaging-derived methods and pathology are summarized in Table 2. In the control cohort, MMG demonstrated poor absolute agreement with pathological DCIS size (ICC = 0.338, 95% CI 0.068–0.561), despite a weak but statistically significant rank correlation (ρ = 0.35, *p* = 0.012).

In the prospective cohort, MMG showed moderate agreement (ICC = 0.693, 95% CI 0.516–0.813), while CEM demonstrated slightly higher agreement (ICC = 0.719, 95% CI 0.553–0.830). In CEM-positive cases, agreement further increased (ICC = 0.745, 95% CI 0.501–0.879).

When we assessed the absolute error for MMG and CEM in the whole prospective cohort, the median absolute error of MMG was 13.4 (IQR 5.57–23.23; 95% CI 10.05–18.10), while the median absolute error of CEM was 15.0 (IQR 6.03–24.25; 95% CI 13.0–19.95). Although the absolute error of the MMG method was slightly lower than that of the CEM method, the Wilcoxon signed-rank test did not show a statistically significant difference between the absolute errors of the two methods (Z = −1.03, *p* = 0.301).

However, when we compared the absolute errors of the two methods analysed in the subgroup of CEM-positive patients (N = 25), we found a slightly but statistically significantly higher accuracy of the CEM method (Z = −2.25, *p* = 0.024; Wilcoxon signed-ranks paired test). In this subgroup of patients, the median absolute error was 18.2 for CEM and 19.95 for MMG, with a Hodges-Lehmann estimate of the median difference of 0.7 (95% CI 0.05–1.30).

Furthermore, Bland–Altman analysis revealed wide limits of agreement across all comparisons, indicating substantial variability at the individual patient level. Mammography showed a small positive median bias, while CEM showed almost zero median bias in the overall cohort but a significant positive bias in CEM-positive patients. Despite the moderate ICC values, the wide limits of agreement indicate that neither modality can reliably predict pathological size at the individual level.

### 3.4. Comparison Between Control Cohort of Patients (MMG-Only) and Prospective CEM Cohort

The clinicopathological characteristics of the study cohorts and their comparison are summarized in Table 1. The two cohorts were comparable in age, laterality, detection mode, hormone receptor status, final pathological DCIS size, surgical specimen size, and reoperation rates. The prospective CEM cohort demonstrated higher BI-RADS categories, larger DCIS size on initial mammography, a higher proportion of high-grade (NG3) DCIS lesions, and a higher Ki-67 proliferation index compared with the control cohort.

Patients in the CEM cohort more frequently showed no residual DCIS on final surgical pathology (8/51 vs. 0/50), whereas the control cohort predominantly presented with residual pure DCIS. A significant linear trend across increasing pathological severity was also observed between cohorts (χ^2^ for trend = 3.99, *p* = 0.046). The addition of CEM was associated with a significant prolongation of the interval from initial MMG to definitive surgery (median 4 vs. 3 months; Hodges–Lehmann median difference 2 months, 95% CI 1–2; *p* < 0.0001).

The distribution of surgical procedures differed significantly between cohorts (*p* = 0.023), with patients in the CEM cohort undergoing more extensive surgical procedures. Reoperation rates were lower in the CEM cohort than in the control cohort (5.9% vs. 17.6%), although this difference did not reach statistical significance (*p* = 0.122). Surgical margin status did not differ significantly between cohorts, with comparable rates of negative margins observed in both groups.

### 3.5. Robustness Analyses of the Association Between Study Group and Reoperation

Multivariable logistic regression adjusting for age, nuclear grade, and treatment center showed no significant association between the study group and reoperation (adjusted OR 0.83, 95% CI 0.15–4.53, *p* = 0.83). A propensity score–adjusted analysis yielded consistent results.

To address potential temporal bias, a sensitivity analysis restricting the control cohort to recent patients only, enrolled between 2022 and 2023, was performed, and to evaluate the impact of missing data, a sensitivity analysis using a missing-indicator approach for nuclear grade was conducted.

Across all analytical approaches, no statistically significant association between study group and reoperation was observed. Effect estimates were consistent in direction but imprecise, with wide confidence intervals.

The consistency of findings across all analyses suggests that the results are not substantially influenced by measured confounding. However, the limited number of outcome events (N = 12) resulted in wide confidence intervals and reduced statistical power; therefore, a modest effect of the intervention cannot be excluded.

### 3.6. Comparison Between CEM-Positive and CEM-Negative Patients

Patients with positive CEM findings had significantly larger DCIS upon final pathological examination compared to those with negative CEM findings. The median difference was 15 mm (95% CI 5–27 mm), and this difference was statistically significant (Mann–Whitney U = 168.0, Z = −2.81, *p* = 0.0049). The time interval between initial mammography and definitive surgery was significantly shorter in patients with positive CEM findings compared with those with negative CEM (median 3.5 vs. 5.0 months). The Hodges–Lehmann median difference was −1 month (95% CI −3 to 0), and this difference was statistically significant (Mann–Whitney U = 198.5, Z = −2.42, *p* = 0.0155).

Although the overall distribution of surgical procedures did not differ significantly between CEM-negative and CEM-positive patients (χ^2^ = 5.52, *p* = 0.238), a significant linear trend was observed toward more extensive surgery in patients with positive CEM findings (χ^2^ for trend = 3.93, *p* = 0.047). All mastectomies occurred in the CEM-positive group. Reoperation rates and surgical margin status did not differ significantly between groups.

When we analysed the association between CEM findings and the histological nuclear grade of DCIS, we found that low-grade DCIS was rare in both groups (only 2 cases total). Contrary to expectations, intermediate-grade DCIS predominated in the CEM-positive group (62.5%), whereas high-grade DCIS was more frequently identified in the CEM-negative cohort (70.6%). There was no statistically significant association between CEM findings and nuclear histological grade of DCIS (χ^2^ = 4.87, *p* = 0.088), and the test for linear trend toward higher nuclear grade with CEM positivity was also not significant (χ^2^ for trend = 3.65, *p* = 0.056). These results are summarized in Table 3.

### 3.7. Impact of CEM on Surgical Planning and Extent, with Pathological Validation

In the overall prospective DCIS cohort (N = 51), contrast-enhanced mammography (CEM) did not significantly change the planned percentage of breast volume resection compared with mammography (MMG) (median 3.8% vs. 8.5%; Hodges–Lehmann median difference −1.6%, 95% CI −5.2 to 4.5; *p* = 0.53, Wilcoxon signed-rank paired test; Table 4).

Similarly, CEM did not significantly alter the proportion of patients exceeding the 15% resection threshold (MMG 33.3% vs. CEM 31.4%; McNemar P = 1.00, Table 5), indicating no overall increase in surgical extent at the population level.

However, among CEM-positive patients (N = 26), CEM significantly increased planned resection volume compared with MMG (median 26.3% vs. 16.1%; Hodges–Lehmann median increase 24.7%, 95% CI 2.0 to 66.9; *p* = 0.021; Wilcoxon signed-rank paired test; Table 4). In this subgroup, CEM increased the planned surgical volume in 18 of 26 patients (69%), while in 7 of 26 (27%), it did not increase or reduce the planned resection. CEM-positive patients also showed a higher rate of ≥15% resections with CEM than with MMG (61.5% vs. 50%), although this difference did not reach statistical significance (McNemar P = 0.45, Table 5).

Correlation between imaging-based surgical planning and pathological DCIS size demonstrated superior accuracy of CEM compared with MMG. In all patients (N = 50), Spearman correlation between planned resection volume and pathological DCIS size was significantly higher for CEM than for MMG (ρ = 0.54 vs. ρ = 0.37, Table 6). This difference was even more pronounced in CEM-positive patients (N = 25), in whom CEM showed a strong correlation with pathological size (ρ = 0.70, Table 6) compared with only a moderate correlation for MMG (ρ = 0.40, Table 6).

**Figure 3:**
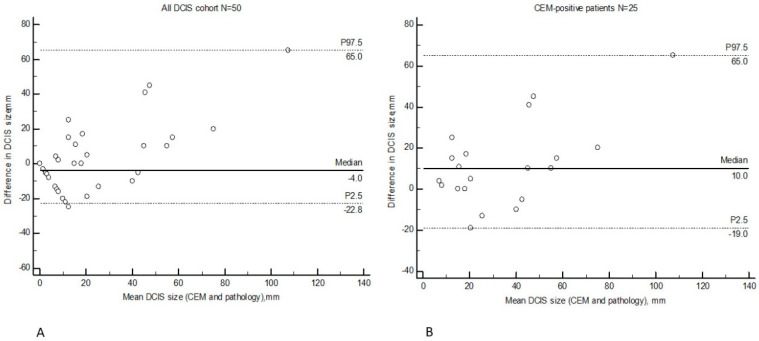
Bland–Altman plots showing agreement between CEM and pathological DCIS size in the overall prospective cohort (**A**) and in CEM-positive patients (**B**). Figure legend: The difference between CEM-derived and pathological DCIS size is plotted against the mean of the two measurements. The x-axis represents the mean DCIS size measured by CEM and pathology, and the y-axis represents the difference between the two measurements (CEM − pathology). The solid horizontal line indicates the median difference (bias), while the dashed lines indicate the non-parametric limits of agreement defined by the 2.5th and 97.5th percentiles. Values below zero indicate underestimation of DCIS extent by CEM relative to pathology. (**A**) Analysis including all patients in the prospective cohort (N = 50). The median bias was −4.0 mm, indicating a slight tendency of CEM to underestimate DCIS size relative to pathology. The limits of agreement ranged from −22.8 to 65.0 mm. (**B**) Analysis restricted to CEM-positive patients (N = 25). The median bias was 10.0 mm, indicating a tendency of CEM to overestimate DCIS size compared with pathology. The limits of agreement ranged from −19.0 to 65.0 mm.

Bland–Altman analysis confirmed these findings, showing systematic underestimation of DCIS extent by MMG in CEM-positive cases, whereas CEM demonstrated near-zero median bias relative to pathology (Figure 3).

**Figure 4:**
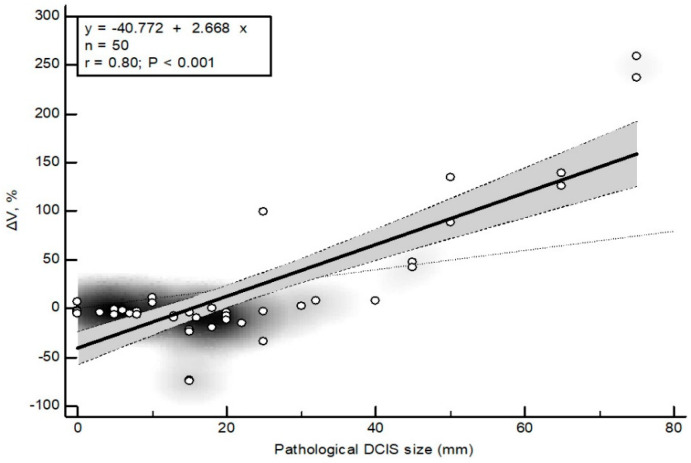
Relationship between pathological DCIS size and CEM-driven change in planned resection volume (ΔV). Figure legend: Scatter plot showing the relationship between pathological DCIS size (x-axis) and the change in planned breast resection volume attributable to contrast-enhanced mammography (ΔV = %V_CEM − %V_MMG; y-axis). Each point represents one patient (N = 50). The solid black line represents the least-squares linear regression fit. The shaded grey band indicates the 95% confidence interval of the regression estimate. The outer dashed lines represent the 95% prediction interval. The background density shading reflects the concentration of data points in regions of higher observation density. One patient from the prospective cohort was excluded from this analysis due to missing pathological measurement of DCIS size. The linear regression analysis demonstrates a strong positive association between pathological DCIS size and CEM-driven surgical escalation (r = 0.80, R^2^ = 0.64, *p* < 0.001), indicating that larger true DCIS size is associated with proportionally greater increases in planned resection volume based on CEM.

To assess whether CEM-driven changes in surgical planning reflected true disease burden, we calculated ΔV as the difference between the percent breast volume resected based on CEM and mammography alone (%V_CEM − %V_MMG). ΔV showed a significant positive correlation with pathologic DCIS size (Spearman ρ = 0.34, *p* = 0.015). Linear regression demonstrated a strong dose–response relationship between DCIS size and CEM-driven surgical escalation (ΔV = −40.8 + 2.67 × DCIS size, R^2^ = 0.64, *p* < 0.0001), indicating that each unit increase in true DCIS size was associated with a 2.7% increase in resection volume attributable to CEM (Figure 4). These findings indicate that CEM increases surgical extent in proportion to actual tumor burden rather than causing nonspecific overestimation.

## 4. Discussion

Ductal carcinoma in situ accounts for approximately 25% of all breast cancer diagnoses. Despite its heterogeneous biological behavior and variable progression risk, standard management remains largely uniform, typically involving surgery followed by radiotherapy and, in selected cases, systemic therapy. In this context, improving preoperative assessment of disease extent is essential not only for diagnostic accuracy but also for enabling more tailored, patient-specific surgical strategies. In the present study, we evaluated the role of contrast-enhanced mammography (CEM) as an adjunct to standard mammography (MMG), with particular emphasis on its potential to support individualized surgical planning.

Our results confirm that MMG provides limited accuracy in estimating DCIS extent, with wide bidirectional discrepancies compared with pathology. In contrast, CEM demonstrated more consistent agreement with pathological size, particularly in CEM-positive cases. Although CEM occasionally underestimated or overestimated lesion size—potentially due to background parenchymal enhancement or tissue shrinkage—it captured disease extent more comprehensively than MMG [23,24,25]. This advantage is especially evident in non-calcified DCIS, where CEM has been shown to achieve a sensitivity of 93.8%, compared with only 43.8% for MMG [26]. Importantly, our findings further indicate that this improved imaging performance is not uniformly distributed across all patients but is most pronounced in lesions exhibiting contrast enhancement, supporting a stratified interpretation of imaging value.

Most prior studies evaluating CEM have focused primarily on diagnostic performance metrics such as sensitivity, lesion detection, or upstaging rates. While these endpoints are important, they do not fully address a central clinical question in modern breast imaging: whether improved detection meaningfully translates into better, more appropriate treatment decisions. A key finding of our study was the relatively high false-negative rate (42%) for DCIS on CEM, with no enhancement observed in 25 of 50 patients. Several factors may explain this observation. First, CEM was performed post-biopsy, and partial lesion removal may have reduced tumor volume below the threshold required for detectable enhancement. Additionally, post-biopsy hematoma or edema may have obscured residual disease or altered local perfusion dynamics, resulting in diminished or absent enhancement. These findings highlight an important limitation: the absence of enhancement on CEM does not reliably exclude the presence of DCIS. Consequently, CEM should not be interpreted in isolation but rather integrated with conventional mammographic findings and clinical context to avoid underestimation of disease.

Importantly, this study extends beyond diagnostic accuracy by evaluating the clinical implications of imaging findings. Although the reoperation rate was lower in the CEM cohort (5.9% vs. 17.6%), this difference did not reach statistical significance, likely due to limited statistical power. Nevertheless, the observed reduction—particularly when compared with historical reoperation rates of 30–40% for DCIS—suggests a potentially meaningful clinical benefit that warrants further investigation [4]. At the same time, the rate of inadequate surgical margins remained unchanged between groups, indicating that the observed differences in reoperation rates cannot be attributed solely to the implementation of CEM. Given that baseline patient characteristics were well balanced, temporal changes in surgical practice must also be considered. Notably, the transition from a strict ≥2 mm margin requirement toward a more conservative, biology-driven approach likely contributed to evolving surgical outcomes during the study period [5,27,28,29].

From a personalized medicine perspective, one of the most relevant findings is that the impact of CEM on surgical planning is not uniform. In CEM-positive patients, CEM frequently resulted in increased planned resection volumes, often exceeding the 15% threshold associated with more complex surgical procedures. In this subgroup, CEM increased the estimated resection volume in 72% of patients, suggesting a substantial influence on surgical decision-making. In contrast, when considering the overall cohort, the net effect of CEM on surgical extent was neutral, with a median change close to zero and wide variability. This divergence indicates that the clinical value of CEM lies not in its routine application to all patients, but in its ability to refine surgical planning in selected subgroups—particularly those with contrast-enhancing lesions.

Importantly, the observed increases in resection volume were proportionate to pathological tumor burden, suggesting that CEM-guided surgical escalation reflects true disease extent rather than unnecessary overtreatment. In this sense, CEM may function as a high-fidelity imaging tool that mitigates the systematic underestimation associated with MMG while avoiding indiscriminate surgical expansion. These findings align with the principles of personalized medicine, where diagnostic tools are used not only to detect disease but to guide patient-specific therapeutic decisions.

At the same time, the integration of CEM into the diagnostic pathway was associated with a longer interval from initial imaging to surgery (median 4 vs. 3 months) and a higher proportion of extensive surgical procedures. These findings are consistent with previous reports indicating that CEM alters surgical management in approximately 20% of patients and leads to mastectomy in a smaller subset [30]. This highlights an important clinical trade-off: while improved delineation of disease extent may enhance surgical precision, it may also increase procedural complexity and delay treatment. Such trade-offs underscore the importance of selective, rather than routine, use of advanced imaging modalities.

**Figure 5:**
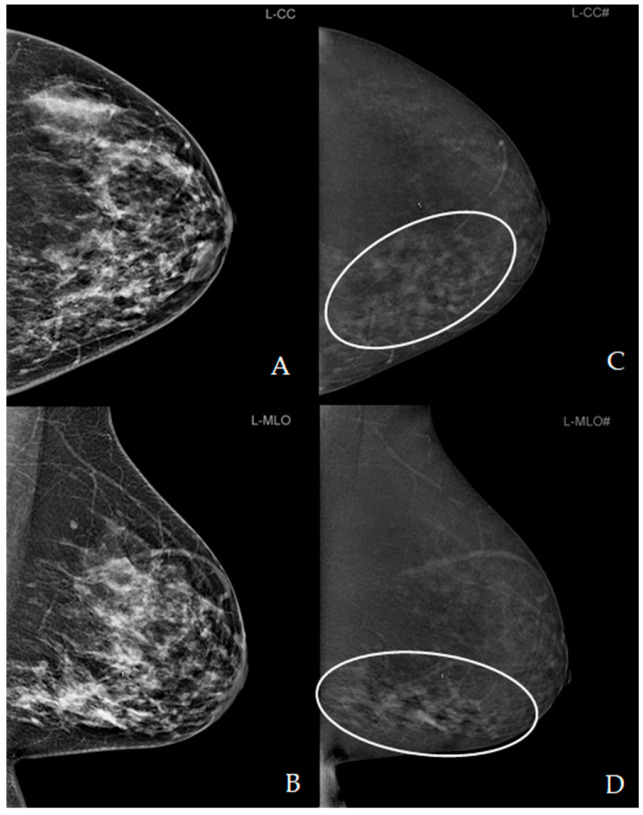
Contrast-enhanced mammography (CEM) of the left breast. Low-energy (LE) images in craniocaudal (**A**) and mediolateral oblique (**B**) projections, equivalent to standard mammography, are shown alongside corresponding recombined images (**C**,**D**). The recombined views demonstrate extensive, diffuse non-mass enhancement (NME) in the lower inner quadrant (white ellipse). In the craniocaudal (CC) projection, the area of enhancement measures approximately 140 × 60 mm. LE images revealed associated clusters of suspicious microcalcifications, which are not clearly visible in this figure.

Our findings further suggest that CEM is more likely to reclassify patients above clinically relevant surgical thresholds, such as the 15% resection volume cut-off (Figure 5). Although this trend did not reach statistical significance, likely due to sample size limitations, it may still have meaningful implications for clinical decision-making. More broadly, these results support a paradigm shift from uniform imaging strategies toward risk-adapted, individualized approaches in which the use of advanced imaging is guided by lesion characteristics and expected clinical impact.

Beyond immediate surgical planning, the long-term oncological implications of CEM-guided management remain to be established. Future studies should investigate whether improved imaging accuracy translates into better local control, reduced recurrence rates, and improved survival outcomes. In addition, a major challenge in contemporary breast oncology remains the differentiation between biologically aggressive (“hazardous”) DCIS and indolent lesions unlikely to progress to invasive disease [31,32]. In this context, imaging modalities such as CEM may contribute not only to anatomical assessment but also to more refined risk stratification.

Importantly, this study was not designed as a predictive model study and therefore does not provide a formal framework for identifying which patients are most likely to benefit from CEM. Rather, the observed differences between CEM-positive and CEM-negative patients should be interpreted as hypothesis-generating and indicative of a heterogeneous treatment effect. Future studies incorporating prospective risk stratification and predictive modeling approaches will be essential to translate these findings into clinically actionable decision-support tools.

The choice between CEM and MRI remains another important consideration. While MRI is widely regarded as the most sensitive modality for breast imaging, its use is limited by cost, availability, examination time, and contraindications. CEM offers a pragmatic alternative, combining functional imaging with greater accessibility and efficiency. Our findings suggest that CEM provides clinically actionable information in selected patients, particularly those with enhancing lesions, although it cannot fully replace MRI in all clinical scenarios.

Before discussing the study limitations, several aspects support the internal validity of the present findings. Imaging interpretation was standardized using consensus reading by experienced breast radiologists, and pathological assessment served as the reference standard. To reduce potential confounding related to the non-randomized design, multivariable, propensity score–adjusted, and sensitivity analyses were performed, all of which yielded consistent results. Nevertheless, residual confounding and temporal bias cannot be fully excluded.

Strengths of the study include its multicentre design, pathology-based validation of imaging findings, and the evaluation of clinically relevant outcomes beyond diagnostic accuracy alone. Unlike most previous studies that primarily focused on lesion detection or size estimation, the present study specifically examined how CEM findings translate into surgical decision-making. A particularly novel aspect of this work is the demonstration that the clinical impact of CEM is heterogeneous rather than universal, with the greatest benefit observed in patients with enhancing lesions. These findings support a more selective and individualized approach to the use of CEM in DCIS management.

This study has several limitations. First, its non-randomized design, combining a prospective interventional cohort with a retrospective control group, introduces potential sources of bias, including temporal and selection effects. Although both cohorts were managed within high-volume institutions with multidisciplinary breast teams and standardized protocols, changes in surgical practice over time cannot be completely excluded. To mitigate this, we performed multivariable, propensity-adjusted, and temporally restricted analyses, all of which yielded consistent results, suggesting that the findings are unlikely to be explained by measured confounding or temporal bias.

Second, the sample size was limited, with a relatively small number of outcome events, resulting in wide confidence intervals and reduced statistical power. As a pilot study, it was not designed to definitively assess clinical endpoints. Post hoc analysis indicated that substantially larger cohorts would be required to detect statistically significant differences in reoperation rates. Therefore, the observed trends should be interpreted cautiously and validated in larger, prospective, multi-centre studies.

Third, although missing data were present, they were limited for key variables and did not materially affect the results in sensitivity analyses.

Finally, surgical decision-making is inherently multifactorial and influenced by factors such as patient preference, surgeon experience, and institutional practice, which cannot be fully captured in quantitative models.

Future research should focus on validating these findings in larger prospective multicentre studies with sufficient statistical power to assess clinically relevant outcomes. In particular, further studies are needed to determine whether the improved estimation of disease extent provided by CEM translates into lower reoperation rates, improved local control, reduced recurrence, and ultimately better long-term oncological outcomes. Given the heterogeneous impact of CEM observed in the present study, future investigations should also aim to develop predictive models and risk-stratification strategies capable of identifying patients most likely to benefit from CEM-guided surgical planning. Such approaches would support a transition from a uniform imaging strategy toward personalized decision-making, in which the use of advanced imaging is tailored according to individual patient and lesion characteristics. Integration of imaging findings with clinicopathological and molecular features may further refine patient selection, improve risk stratification, and facilitate more individualized treatment planning. In the broader context of personalized medicine, future studies should explore whether CEM can help distinguish patients who may benefit from more extensive surgery from those for whom treatment de-escalation may be appropriate, thereby optimizing the balance between oncological safety and treatment burden. Finally, comparative studies evaluating CEM alongside breast MRI, including analyses of cost-effectiveness, accessibility, patient burden, and clinical outcomes, will be important to better define the optimal role of CEM within contemporary DCIS management pathways.

## 5. Conclusions

The findings of this study demonstrate that CEM improves the estimation of DCIS extent and supports more individualized, risk-adapted surgical planning, particularly in patients with enhancing lesions. While no significant differences in surgical outcomes were observed in this pilot cohort, the findings highlight the potential of CEM to contribute to personalized management strategies in DCIS. Further prospective studies are needed to validate its role in clinical decision-making.

## Figures and Tables

**Table 1:** Study cohorts: clinicopathological characteristics and their comparison.

Characteristic	Control Cohort N = 51	Prospective CEM Cohort N = 51	*p* Value
Age (years) Median (range)	64 (44–86)	65 (45–84)	0.786 ^¶^
**Age, N (%)**			
≤60 years	14 (27.5)	15 (29.4)	0.831 ^§^
>60 years	37 (72.5)	36 (70.6)	
**Laterality, N (%)**			
Right breast	27 (52.9)	24 (48.0)	0.692 ^§^
Left breast	24 (47.1)	26 (52.0)	
**Detection, N (%)**			
Screening	17 (33.3)	18 (36.0)	0.836 ^§^
Other	34 (66.7)	32 (64.0)	
**MMG BI-RADS classification, N (%)**			
BI-RADS 0	0 (0)	14 (28.0)	<0.0001 ^±^
BI-RADS 1	0 (0)	0 (0)	
BI-RADS 2	0 (0)	0 (0)	
BI-RADS 3	9 (17.6)	0 (0)	
BI-RADS 4	37 (72.5)	25 (50.0)	
BI-RADS 5	5 (9.8)	11 (22.0)	
**DCIS size MMG (mm) Median (range)**	11 (2–50)	16 (3–120)	0.011 ^¶^
**Clinical examination, N (%)**			
Not performed	0 (0)	8 (15.7)	0.006 ^±^
No physical signs	50 (98.0)	40 (78.4)	
Probably benign	0 (0)	0 (0)	
Suspicious of malignancy	1 (2.0)	3 (5.9)	
**ER, N (%)**			
Negative (<1%)	7 (13.7)	8 (15.7)	1.00 ^§^
Positive (≥1%)	44 (86.3)	43 (84.3)	
**PR, N (%)**			
Negative (<1%)	15 (29.4)	21 (41.2)	0.223 ^§^
Positive (≥1%)	36 (70.6)	30 (58.8)	
**Ki-67 (%) Median (range)**	16 (3–50)	20 (3–41)	0.032 ^¶^
**Ki-67, N (%)**			
≤30%	48 (94.1)	28 (77.8)	0.045 ^§^
>30%	3 (5.9)	8 (22.2)	
**Final pathology report, N (%)**			
No residual DCIS	0 (0)	8 (15.7)	0.016 ^±^
DCIS	47 (92.1)	41 (80.4)	
DCIS + microinvasive ca	3 (5.9)	1 (2.0)	
DCIS/LCIS + invasive ca	0 (0)	1 (2.0)	
**Type of in situ disease, N (%)**			
DCIS	44 (86.3)	50 (98.0)	0.259 **^±^**
Papillary DCIS	2 (3.9)	0 (0)	
LCIS	2 (3.9)	1 (2.0)	
**Nuclear grade of DCIS, N (%)**			
1	0 (0)	2 (3.9)	0.007 ^±^
2	43 (89.6)	32 (62.7)	
3	5 (10.4)	17 (33.3)	
**Final pathological DCIS size (mm) Median (range)**	14 (1–70)	15 (0–75)	0.719 ^¶^
**Time (months) interval from initial MMG to surgery, median (range)**	3 (1–13)	4 (1–11)	<0.0001 ^¶^
**Surgical specimen size (mm) median (range)**	73.5 (10–145)	70 (30–270)	0.997 ^¶^
**Surgical procedure, N (%)**			
Excisional biopsy	3 (5.9)	5 (9.8)	0.023 ^±^
Wide resection/segmentectomy	42 (82.4)	27 (52.9)	
Quadrantectomy	5 (9.8)	14 (27.5)	
Subcutaneous mastectomy	0 (0)	3 (5.9)	
Mastectomy	1 (2.0)	2 (3.9)	
**Reoperation, N (%)**			
No	42 (82.4)	48 (94.1)	0.122 ^§^
Yes	9 (17.6)	3 (5.9)	
**Re-Reoperation, N (%)**			
No	49 (96.1)	51 (100)	0.248 ^§^
Yes	2 (3.9)	0 (0)	
**Surgical margin status, N (%)**			
Negative free margin, DCIS ≥ 2 mm	34 (68.0)	28 (57.1)	0.629 **^±^**
Negative but close to margin, DCIS < 2 mm	11 (22.0)	13 (26.5)	
Positive, DCIS focally on inked margin	3 (6.0)	6 (12.2)	
Positive, DCIS wide on inked margin	2 (4.0)	2 (4.2)	
**Surgical margin status, N (%)**			
Negative	45 (90.0)	41 (83.7)	0.385 **^§^**
Positive	5 (10.0)	8 (16.3)	

^¶^ Mann–Whitney test; ^§^ Fisher’s exact test; ^±^ Chi-squared test. ER = estrogen receptor; PR = progesterone receptor. Missing data: Ki-67 unavailable for 15 patients (institutional practice at UHC Zagreb); pathological DCIS size missing in 2 patients (1 per cohort); nuclear grade missing in 3 patients (control cohort); surgical margin status missing in 3 patients (1 control, 2 prospective).

**Table 2:** Agreement between imaging-derived and pathological DCIS size: Spearman correlation, intraclass correlation coefficient (ICC), and Bland–Altman analysis.

Cohort	Imaging	Reference Standard	N	Spearman’s ρ	*p* Value	ICC * (95%CI)	Median Bias (mm) *	95% CI	Lower LoA (mm)	Upper LoA (mm)
Control	MMG	Pathological DCIS size	50	0.35	0.012	0.338 (0.068–0.561)	+3.0	−1.40 to 6.0	−24.75	+37.50
Prospective CEM	MMG	Pathological DCIS size	50	0.37	0.008	0.693 (0.516–0.813)	+3.0	0.21 to 8.59	−36.5	+46.25
	CEM (full cohort)	Pathological DCIS size	50	0.54	<0.001	0.719 (0.553–0.830)	−4.0	−11.19 to 0.00	−22.75	+65.00
	CEM (positive only)	Pathological DCIS size	25	0.67	<0.001	0.745 (0.501–0.879)	+10.0	0.29 to 19.56	−19.0	+65.00

* ICC calculated using a two-way model for absolute agreement and single measures. Bland–Altman analysis was performed using a non-parametric approach where bias is reported as the median difference (imaging − pathology) and the limits of agreement (LoA) are defined by the 2.5th (lower LoA) and 97.5th (upper LoA) percentiles of the differences.

**Table 3:** Prospective CEM cohort: comparison between CEM-positive and CEM-negative patients.

Characteristic	CEM-Negative N = 25	CEM-Positive N = 26	*p* Value
Age (years) Median (range)	68 (53–83)	64 (45–84)	0.111 ^¶^
**Age, N (%)**			
≤60 years	6 (24.0)	9 (34.6)	0.541 ^§^
>60 years	19 (76.0)	17 (65.4)	
**Laterality, N (%)**			
Right breast	15 (60.0)	9 (34.6)	0.095 ^§^
Left breast	10 (40.0)	17 (65.4)	
**Detection, N (%)**			
Screening	10 (40.0)	8 (30.8)	0.565 ^§^
Other	15 (60.0)	18 (69.2)	
**MMG BI-RADS classification, N (%)**			
BI-RADS 0	6 (25.0)	8 (30.8)	0.859 ^±^
BI-RADS 1	0 (0)	0 (0)	
BI-RADS 2	0 (0)	0 (0)	
BI-RADS 3	0 (0)	0 (0)	
BI-RADS 4	13 (54.2)	12 (46.2)	
BI-RADS 5	5 (20.8)	6 (23.1)	
**DCIS size MMG (mm) median (range)**	15 (4.4–50.0)	24 (3–120)	**0.015 ^¶^**
**Clinical examination, N (%)**			
Not performed	4 (16.0)	4 (15.4)	0.339 ^±^
No physical signs	21 (84.0)	19 (73.1)	
Suspicious of malignancy	0 (0)	3 (11.5)	
**ER, N (%)**			
Negative (<1%)	5 (20.0)	3 (11.5)	0.465 ^§^
Positive (≥1%)	20 (80.0)	23 (88.5)	
**PR, N (%)**			
Negative (<1%)	13 (52.0)	8 (30.8)	0.159 ^§^
Positive (≥1%)	12 (48.0)	18 (69.2)	
**Ki-67 (%) Median (range)**	20 (5–40)	20 (3–41)	0.774 ^¶^
**Ki-67, N (%)**			
≤30%	14 (73.7)	14 (82.4)	0.695 ^§^
>30%	5 (26.3)	3 (17.6)	
**Final pathology report, N (%)**			
No residual DCIS	4 (16.0)	4 (15.4)	0.381 ^±^
DCIS	21 (84.0)	20 (76.9)	
DCIS + microinvasive ca	0 (0)	1 (3.8)	
DCIS/LCIS + invasive ca	0 (0)	1 (3.8)	
**Type of in situ disease, N (%)**			
DCIS	25 (100)	25 (96.2)	0.327 ^±^
Papillary DCIS	0 (0)	0 (0)	
LCIS	0 (0)	1 (3.8)	
**Final pathological DCIS size (mm) median (range)**	15 (0–25)	25 (0–75)	**0.005 ^¶^**
**Time (months) interval from initial MMG to surgery, median (range)**	5 (1–11)	3.5 (1–8)	**0.016 ^¶^**
**Surgical specimen size (mm) median (range)**	51 (30–100)	85 (30–270)	**0.005 ^¶^**
**Nuclear grade of DCIS, N (%)**			
**1**	1 (4.0)	1 (3.8)	0.088 ^±^
**2**	12 (48.0)	20 (76.9)	
**3**	12 (48.0)	5 (19.2)	
**Reoperation, N (%)**			
No	24 (96.0)	24 (92.3)	1.00 ^§^
Yes	1 (4.0)	2 (7.7)	
**Surgical margin status, N (%)**			
Negative free margin, DCIS ≥ 2 mm	13 (56.5)	15 (57.7)	0.447 ^±^
Negative but close to margin, DCIS < 2 mm	5 (21.7)	8 (30.8)	
Positive, DCIS focally on inked margin	3 (13.0)	3 (11.5)	
Positive, DCIS wide on inked margin	2 (8.7)	0 (0)	
**Surgical margin status, N (%)**			
Negative	18 (78.3)	23 (88.5)	0.448 ^§^
Positive	5 (21.7)	3 (11.5)	

^¶^ Mann–Whitney test; ^§^ Fisher’s exact test; ^±^ Chi-squared test. ER = estrogen receptor; PR = progesterone receptor.

**Table 4:** Impact of CEM on planned resection volume (continuous analysis).

Population	Median %V MMG	Median %V CEM	Hodges–Lehmann Δ	95% CI	*p* Value *
**All DCIS cohort (N = 51)**	8.5	3.8	–1.6	–5.2 to +4.5	0.53
**CEM-positive (N = 26)**	16.1	26.3	+24.7	+2.0 to +66.9	0.021

Median %V MMG = Percentage volume resection according to MMG; Median %V CEM = Percentage volume resection according to CEM; Hodges–Lehmann Δ = Hodges-Lehmann median difference; CI = Confidence interval; * Wilcoxon signed-rank paired test.

**Table 5:** Impact of CEM on crossing the 15% surgical threshold.

Population	MMG > 15%	CEM > 15%	Net Change	McNemar P
**All DCIS cohort (N = 51)**	33.3%	31.4%	–1.9%	1.00
**CEM-positive (N = 26)**	50.0%	61.5%	+11.5%	0.45

**Table 6:** Correlation between imaging-based planning and pathology.

Population	Method	Spearman ρ	*p* Value
**All DCIS cohort (N = 50)**	MMG vs. pathology	0.37	0.009
	CEM vs. pathology	0.54	<0.001
**CEM-positive (N = 25)**	MMG vs. pathology	0.40	0.046
	CEM vs. pathology	0.70	<0.001

## Data Availability

All data generated or analysed during this study are included in this article. Further enquiries can be directed to the corresponding author.

## Abbreviations

The following abbreviations are used in this manuscript:

DCIS	Ductal carcinoma in situ
CEM	Contrast-enhanced mammography
MMG	Mammography
MRI	Magnetic resonance imaging
LoA	Limits of agreement
ΔV	Relative change in planned resection volume
SLNB	Sentinel lymph node biopsy
ASCO	American Society of Clinical Oncology
SSO	Society of Surgical Oncology
ASTRO	American Society for Radiation Oncology
PACS	Picture Archiving and Communication System
MLO	Mediolateral oblique (view)
CC	Craniocaudal (view)
VABB	Vacuum-assisted breast biopsy
CNB	Core needle biopsy
FNR	False negative rate
R^2^	Coefficient of determination
ICC	Intraclass correlation coefficient
BCS	Breast-conserving surgery
LCIS	Lobular carcinoma in situ
pLCIS	Pleomorphic Lobular carcinoma in situ
CHC	Clinical Hospital Centre Rijeka
UHC	University Hospital Centre Zagreb
STARD	Standards of Reporting of Diagnostic Accuracy
ER	estrogen receptor
PR	progesterone receptor
ρ	Spearman’s rank correlation coefficient
IQR	Interquartile ranges
BI-RADS	Breast Imaging Reporting and Data System
